# Multimodal image analysis of clinical influences on preterm brain development

**DOI:** 10.1002/ana.24995

**Published:** 2017-08-19

**Authors:** Gareth Ball, Paul Aljabar, Phumza Nongena, Nigel Kennea, Nuria Gonzalez‐Cinca, Shona Falconer, Andrew T.M. Chew, Nicholas Harper, Julia Wurie, Mary A. Rutherford, Serena J. Counsell, A. David Edwards

**Affiliations:** ^1^ Centre for the Developing Brain, King's College London, St Thomas' Hospital London United Kingdom; ^2^ St Georges, University of London, St Georges Hospital London United Kingdom; ^3^ Developmental Imaging, Murdoch Childrens Research Institute, The Royal Children's Hospital Melbourne Victoria Australia

## Abstract

**Objective:**

Premature birth is associated with numerous complex abnormalities of white and gray matter and a high incidence of long‐term neurocognitive impairment. An integrated understanding of these abnormalities and their association with clinical events is lacking. The aim of this study was to identify specific patterns of abnormal cerebral development and their antenatal and postnatal antecedents.

**Methods:**

In a prospective cohort of 449 infants (226 male), we performed a multivariate and data‐driven analysis combining multiple imaging modalities. Using canonical correlation analysis, we sought separable multimodal imaging markers associated with specific clinical and environmental factors and correlated to neurodevelopmental outcome at 2 years.

**Results:**

We found five independent patterns of neuroanatomical variation that related to clinical factors including age, prematurity, sex, intrauterine complications, and postnatal adversity. We also confirmed the association between imaging markers of neuroanatomical abnormality and poor cognitive and motor outcomes at 2 years.

**Interpretation:**

This data‐driven approach defined novel and clinically relevant imaging markers of cerebral maldevelopment, which offer new insights into the nature of preterm brain injury. Ann Neurol 2017;82:233–246

Preterm birth is a leading cause of infant mortality and morbidity in economically developed countries.^1^ Preterm infants carry a profound risk of severe neurological disability along with a range of deficits across several domains, including cognition, motor coordination, and behavior.^2^, ^3^ It is likely that long‐term neurodevelopmental outcomes following preterm birth are predicated on early alterations to brain development, and the identification of modifiable risk factors at an early time point has the potential to improve continued clinical management in the preterm population.^4^, ^5^

Preterm brain injury is a multifactorial disorder encompassing alterations to both cortical and subcortical gray matter, as well as white matter.^6^ Quantitative magnetic resonance (MR) image (MRI) analysis has shown that, even in the absence of major focal destructive lesions, there are alterations across all tissue compartments following preterm birth, including: reduced white matter anisotropy^7^; altered cortical volume and complexity^8^, ^9^; tissue loss and microstructural disorganisation in the basal ganglia^10^, ^11^; and ventricular enlargement.^12^ The clinical significance of these observations is uncertain and clouded by the complex impact of genetic, environmental, and clinical factors. Aside from gestational age at birth, antenatal conditions, such as chorioamnionitis^13^ and intrauterine growth restriction (IUGR)^14^ and male sex,^15^ familial factors, such as socioeconomic risk,^16^ and postnatal clinical risk factors, including the need for respiratory support^17^ and early nutrition,^18^ have all been identified as potential risk factors in this population.

Most previous etiological research has focused upon the impact of specific neurological abnormalities: focal destructive brain lesions such as hemorrhagic parenchymal infarction (HPI) and periventricular leucomalacia (PVL). Although the clinical importance of focal lesions is well established,^19^ the relative low incidence of HPI and PVL cannot explain the significant neurocognitive impairment observed in survivors of preterm birth. In contrast, diffuse MRI signal abnormalities are highly prevalent in preterm infants at term‐equivalent age,^19^ but do not provide sensitive or specific prognostis of clinical outcomes.^20^ It is clear that the current clinicopathological model of preterm brain injury is incomplete.

Selection of particular imaging markers a priori can bias observations and discards the rich data available from multiple imaging modalities. New approaches to data‐driven image analysis allow for nonsubjective analysis of multimodal data sets to discover new imaging markers and their relation to clinical events and outcomes.

In this study, we use data‐driven, multivariate methods to test the hypothesis that brain development is altered by multiple environmental factors interacting with early extrauterine exposure following preterm birth. We identified specific relationships between the perinatal environment and the brain by drawing on a large, prospective cohort of preterm infants that underwent a comprehensive multimodal MRI examination at term‐equivalent age. Specifically, we combined independent component analysis (ICA) and canonical correlation analysis (CCA) to identify associations between a set of clinical/environmental risk factors and brain development in preterm infants defined using T1‐weighted (T1w), T2‐weighted (T2w), and diffusion MRI. We found a set of novel patterns of brain abnormality associated with specific clinical risk factors and correlated with cognitive and motor outcomes at 2 years.

## Patients and Methods

### Participants

Infants were recruited from the ePrime randomized, controlled trial of MRI and cranial ultrasound (NCT01049594). This research was approved by the Hammersmith, Queen Charlotte's, and Chelsea Research Ethics Committee (09/H0707/98), and written parental consent was obtained for each infant before imaging. Infants were born before 33 weeks gestation between April 2010 and April 2013, and MRI was performed at term‐equivalent age. Exclusion criteria included: past MR imaging, major congenital malformations, and the presence of metallic implants.

Of 511 infants, 61 were excluded because of unavailable or motion‐artefacted MRI and 1 withdrew. The final study population consisted of 449 infants (226 male) with a median gestational age at birth of 30^+1^ weeks (range, 23^+4^–32^+6^) and a median postmenstrual age at scan of 42^+4^ weeks (range, 37^+6^–55^+2^). After radiological assessment, parenchymal lesions likely to confer a high risk of poor outcome were identified in 37 infants included in the study (8%). These included cystic PVL (n = 7), HPI (n = 6 left; n = 5 right), hemorrhage (cerebellar, n = 4; occipital, n = 2), multiple/extensive white matter lesions (n = 10), posthemorrhagic ventricular dilatation (n = 1), large temporal lobe cyst (n = 1), and periventricular cyst (n = 1).

### Procedures

#### MRI Acquisition

MRI was performed on a Philips 3‐Tesla system (Philips Medical Systems, Best, The Netherlands) within the Neonatal Intensive Care Unit using an eight‐channel phased array head coil.

T1w MRI was acquired using: repetition time (TR): 17ms; echo time (TE): 4.6ms; flip angle: 13 degrees; slice thickness: 0.8mm; field of view: 210mm; and matrix: 256 × 256 (voxel size, 0.82 × 0.82 × 0.8mm). T2w fast‐spin echo MRI was acquired using: TR: 14,730ms; TE: 160ms; flip angle: 90 degrees; field of view: 220mm; and matrix: 256 × 256 (voxel size, 0.86 × 0.86 × 2mm) with 1mm overlap. Single‐shot echo‐planar diffusion weighted imaging was acquired in the transverse plane in 32 noncollinear directions with a single B0 volume using the following parameters: TR: 8 000ms; TE: 49ms; slice thickness: 2mm; field of view: 224mm; matrix: 128 × 128mm (voxel size, 1.75 × 1.75 × 2mm); *b*‐value: 750s/mm^2^; and sensitivity encoding factor of 2.

Pulse oximetry, temperature, and heart rate were monitored throughout, and ear protection was used for each infant (President Putty; Coltene Whaledent, Mahwah, NJ; MiniMuffs; Natus Medical Inc, San Carlos, CA).

#### Image Processing

T1w and T2w images were brain‐extracted (FSL's Brain Extraction Tool; FSL 5.0.8; http://fsl.fmrib.ox.ac.uk/fsl) and corrected for bias field inhomogeneities.^21^ Each subject's T1w image was aligned to an age‐appropriate template^22^ using nonlinear registration.^23^ Voxel‐wise maps of volume change induced by the transformation were characterized by the determinant of the Jacobian operator, referred to here as the Jacobian map. Each map was log‐transformed so that values greater than 0 represent local areal expansion in the subject relative to the target and values less than 0 represent areal contraction. Before transformation into template space, T2w tissue intensities were first matched to the population‐based T2w template using a piece‐wise linear transform^24^ to allow for quantitative comparison across subjects. T2w images were then aligned to the corresponding T1w images with rigid‐body registration and transformed into template space using the previously calculated deformations. T1w‐derived Jacobian maps and T2w intensity images were iteratively smoothed to a full width at half maximum (FWHM) of 8mm (AFNI's 3dBlurToFWHM; http://afni.nimh.nih.gov/afni) before linked independent component analysis (ICA).

Diffusion data were visually assessed and gradient volumes removed if affected by motion‐induced slice dropout artefacts. In total, 31.8% (143 of 449) of subjects had at least one gradient volume removed (mean, 2.35; range, 1–9). Motion and eddy current correction was then performed by aligning all diffusion volumes to the reference *b* = 0 image and the corresponding *b*‐vectors rotated accordingly. Diffusion tensors were modeled at each voxel using a weighted least squares fit to derive maps of fractional anisotropy (FA) and mean diffusivity (MD) for each subject.

Skeletonized white matter FA maps were produced following the tract‐based spatial statistics protocol.^25^ FA maps were aligned to a study‐specific template and averaged to create a mean FA map. The mean map was skeletonized and maximal FA values from nearby voxels in the individual, aligned maps projected to the skeleton. An analogous approach (gray‐matter–based spatial statistics) was used to create skeletonized cortical mean diffusivity maps.^8^ MD maps were aligned to a study‐specific T2w template, alongside probabilistic cortical segmentations derived from the corresponding T2w images.^26^ Mean diffusivity values from voxels with maximal cortical probability in the aligned cortical segmentation maps were projected onto a mean cortical skeleton to create skeletonized MD maps. In addition, in order to include microstructural measures from the deep gray matter, a set of atlas‐defined deep gray matter labels^26^ was applied to the mean diffusivity maps after transformation into template space. MD within the masks was smoothed to 5mm FWHM and added to the cortical skeleton to provide spatial maps of both cortical and deep gray matter MD for analysis.

#### Linked ICA

Linked ICA was used to provide a data‐driven, unsupervised approach for dimension reduction and multimodal data analysis that identifies correlated patterns of variation across multiple imaging modalities.

**Figure 1:**
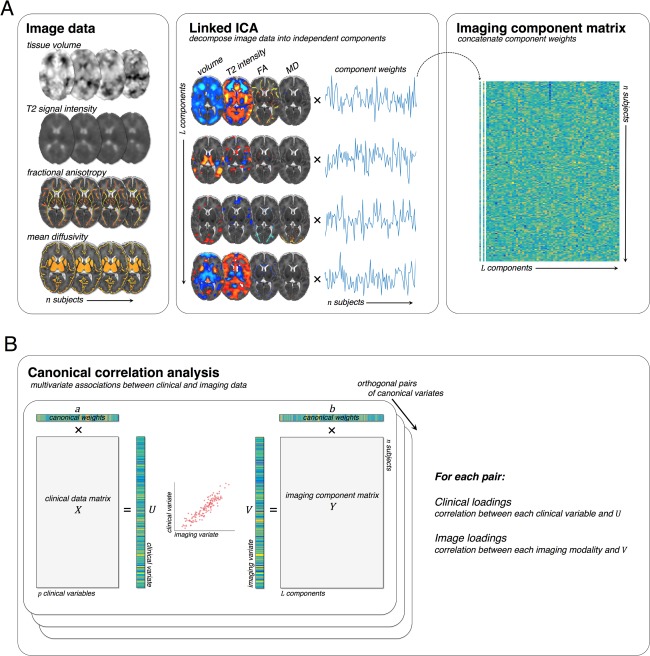
Analysis pipeline. Multimodal imaging data sets were decomposed into a set of linked independent components (A). Each component represents a spatially independent pattern of variation linked across modalities by a shared subject course, or component weight. Subject‐specific weights for all components were concatenated before CCA. The canonical correlation analysis is illustrated in (B). Multivariate associations between clinical and imaging data are sought by calculating model weights, *a* and *b*, that maximize the correlation between the clinical and imaging variates, *U* and *V*. Subsequent canonical pairs are sought under that constraint that they are orthogonal. Clinical and imaging loadings are calculated by correlating the canonical variates with the original clinical and imaging data sets. CCA = canonical correlation analysis; FA = fractional anisotropy; ICA = independent component analysis; MD = mean diffusivity.

An overview of this process is presented in Figure 1A. Individual images are concatenated into four modality groups. The imaging data are then decomposed into a set of multimodal components. Each component comprises a set of spatially independent maps, one per imaging modality, linked together by a single shared subject course, or component weight. The component weight describes the extent to which the linked patterns of variation described by each component map are expressed in each subject's multimodal data and can be used as a parsimonious representation of the full data set.

We used FSL's Linked ICA (FLICA) toolbox implemented in Matlab (R2013a; The MathWorks, Inc., Natick, MA)^27^ to decompose our multimodal data set into imaging components. Within a Bayesian framework, linked ICA is robust to differing units, signal‐ and contrast‐to‐noise ratios, image size, and image smoothness across modalities and incorporates automatic relevance determination to allow the rejection of components unsupported by the data.^27^ For details on the implementation and estimation of this model, we direct interested readers to the work of Groves et al.^27^, ^28^

Before linked ICA, variance normalisation was performed separately on each set of aligned image maps.^29^ Linked ICA was then run on the full data set (449 subjects × 4 modalities) for 5,000 iterations with an upper limit of 100 components specified. Four components were rejected, leaving a total of 96 imaging components.

### Clinical Data

Clinical information including maternal/familial factors (eg, social deprivation score, mother's age), antenatal factors (including chorioamnionitis and intrauterine growth restriction), and neonatal factors (such as gestational age at birth) was collected as part of the clinical trial. All factors that were complete or near complete for all infants were included for analysis. Clinical factors entered into the model are shown in Table 1.

**Table 1:** Clinical Characteristics of the Cohort

		Data	Missing Cases (%)
**Birth Characteristics**			
Gestational age at birth (range)/weeks		30^+1^ (23^+4^–32^+6^)	
Birth weight (range)/g		1,305 (552–2,600)	
Male (%)		226 (50.33)	
Nonsingleton (%)		144 (32.07)	
**Maternal Complications**			
Pregnancy‐associated hypertension (%)		83 (18.49)	
Hypertension		22 (4.89)	
Premature rupture of membranes (%)		72 (16.04)	
Antenatal hemorrhage (%)		59 (13.14)	
Accidental hemorrhage (%)		17 (3.79)	
Urinary tract infection (%)		11 (2.45)	
Gestational diabetes (%)		20 (4.45)	
Oligohydramnios (%)		34 (7.57)	
Polyhydramnios (%)		2 (0.45)	
Drug abuse (%)		6 (1.33)	
In vitro fertilization (%)		65 (14.47)	
Bacterial infection (%)		26 (5.79)	
**Clinical Complications**			
Birth by Caesarean section (%)		292 (65.03)	
Elective Caesarean (%)		39 (8.69)	
Twin‐twin transfusion (%)		9 (2.00)	
Choriamnionitis (%)		30 (6.68)	
Intrauterine growth restriction (%)		66 (14.70)	
Incomplete steroid administration (%)		68 (15.14)	
Surfactant administration (%)		237 (52.78)	
Mechanical ventilation (range)/days		2.89 (0–65)	
CPAP (range)/days		16.74 (0–97)	
Total parenteral nutrition (range)/days		8.92 (0–151)	1 (0.23)
Patent ductus arteriosus (%)		23 (5.12)	
Surgical treatment for necrotising enterocolitis (%)		9 (2.00)	
Formula feeding (%)		256 (60.38)	
No maternal breast milk (%)		62 (14.62)	
**Familial Characteristics**			
Index of multiple deprivation (range)		17.57 (1.73–60.58)	
Mother's age at birth/years (range)		32.75 (16.63–53.42)	

CPAP = continuous positive airway pressure.

### Neurodevelopment Outcome

Of 449 participants, follow‐up assessments at 18‐24 months corrected age were available for n = 425. For these infants, neurodevelopmental outcome was assessed using the Bayley Scales of Infant and Toddler Development, Third Edition (BSID‐III) administered by a specialist psychologist and neurodevelopmental pediatrician. Composite cognitive, language, and motor scores are reported as markers of outcome.

### Statistical Analysis

In order to investigate the relationship between clinical and imaging data, we performed canonical correlation analysis (CCA). CCA seeks to maximize the correlation between successive linear transformations of two variable sets, *X* and *Y* (Fig 1B). The result is a *canonical correlation* between two *variates, U=aX* and *V=bY*, where *a* and *b* are the *canonical vectors* or *weights* sought by the model. Once a pair of canonical variates is found, a successive pair is sought subject to the constraint that they are uncorrelated with the first pair, and so on.

Here, we enter the component weights derived from linked ICA (Fig 1A) alongside a set of clinical and environmental variables (Table 1) into CCA to identify multivariate clinical‐image pairs. The statistical significance of the correlation between canonical pairs was assessed sequentially with a permutation test, swapping the rows of one feature matrix with respect to the other 10,000 times and recording the maximum correlation between pairs. Canonical correlation analysis was performed using Scikit‐learn 0.17.^30^

To determine the relationship between each clinical variable in *X* and the model, we calculate the *loading*, or correlation, between the original variable (e.g: *X*_*1*_,…,*X*_*n*_) and their respective canonical variate (*U*_*1*_,…,*U*_*m*_) in each pair, where *n* is the number of original variables in *X* and *m* is the number of canonical pairs. In each case, loading strength was assessed with permutation testing (10,000 permutations). Note that the canonical *weights* for each pair show the *unique* contribution of each variable to the synthetic canonical variate whereas *loadings* show the overall correlation; hence, variables with positive canonical *loadings* may still have negative *coefficients* that reflect a dependence on, or interaction with, other contributing variables. To estimate confidence intervals (CIs) for canonical correlations, weights, and loadings, we implemented a bootstrapping procedure, resampling our data with replacement 10,000 times and fitting the CCA model to each sample.

In order to visualize the imaging phenotype associated with each clinical covariate, we performed an analogous procedure, estimating voxel‐wise loadings by calculating correlations between the original imaging data sets and each canonical variate (*V*_*1*_,…,*V*_*m*_). Loading maps were assessed for significance using permutation testing and corrected for multiple comparisons across voxels using FSL's *randomise* tool (http://fsl.fmrib.ox.ac.uk/fsl).

Associations between the canonical variates in each pair and neurodevelopmental outcome were assessed using linear regression (SPSS v21; IBM Corp., Armonk, NY).

### Multimodal Interactive Maps

To allow detailed exploration of all significant imaging‐clinical pairs, we have made available interactive statistical maps for each imaging modality to view online at NeuroVault (http://neurovault.org/collections/2178).

## Results

**Figure 2:**
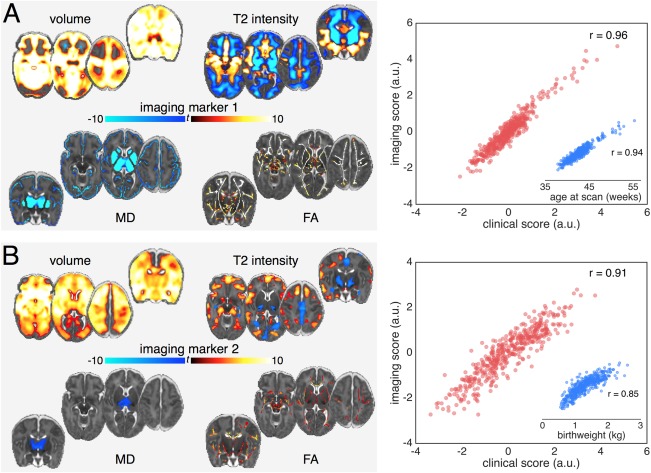
Imaging patterns correlating with age at scan and low birth weight. Image loadings associated with the first two canonical pairs (A: pair 1; B: pair 2). Maps are shown thresholded at *p* < 0.01 (corrected for multiple comparisons), the colour bar indicates the *t*‐statistic. Warm colours show regions positively correlated with the canonical variate, and *vice versa*. The correlation between the canonical imaging and clinical variates for each pair are shown in the scatterplot in red. Inset: scatterplot (blue) of the correlation between the imaging variate score and the clinical factor with the largest clinical variate loading. Unthresholded statistical maps are available to view online at http://neurovault.org/collections/2178. a.u. = arbitrary units; FA = fractional anisotropy; MD = mean diffusivity.

**Figure 3:**
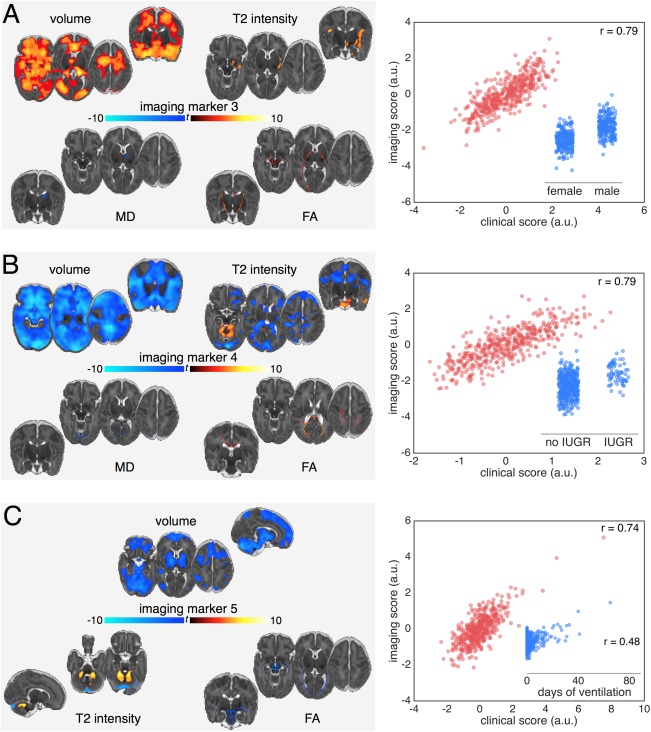
Imaging patterns correlating with male sex, intrauterine growth restriction, and postnatal illness. Imaging markers for canonical pairs 3 (A), 4 (B) and 5 (C). Images are displayed as in Figure 2. Modalities are only displayed if any voxels showed a significant association with the corresponding imaging variate (*p* < 0.01). Unthresholded statistical maps are available to view online at http://neurovault.org/collections/2178. a.u. = arbitrary units; FA = fractional anisotropy; MD = mean diffusivity.

CCA revealed five significant paired relationships between clinical and imaging data within the preterm cohort (*p* < 0.05; 10,000 permutations). Table 2 shows the canonical correlations (with 95% CI) between the first 10 pairs. For each significant pair, Figures 2 and 3 illustrate the imaging loadings, the strength of association between the imaging and clinical variate scores, and the association between the imaging variate and the principal clinical variable (ie, the clinical factor with the maximal canonical loading). Loadings and weights showing the correlation and unique contribution to the canonical pair of the clinical variables with the highest loadings are shown in Table 3.

**Table 2:** Canonical Correlations

		95% Confidence Intervals		
Canonical Pair	Canonical Correlation	Lower	Upper	*p*
**1**	**0.959**	**0.894**	**0.958**	**0.0001** a
**2**	**0.910**	**0.883**	**0.928**	**0.0001** a
**3**	**0.792**	**0.739**	**0.834**	**0.013** a
**4**	**0.785**	**0.724**	**0.821**	**0.013** a
**5**	**0.740**	**0.651**	**0.802**	**0.018** a
6	0.698	0.604	0.762	0.077
7	0.674	0.602	0.737	0.473
8	0.640	0.573	0.711	0.993
9	0.623	0.541	0.694	1.000
10	0.604	0.535	0.688	1.000

a Significant at *p* < 0.05 after 10,000 permutations.

**Table 3:** Canonical Loadings and Weights for Clinical Variables in Each Canonical Pair, Sorted by Loading

Clinical Variable		95% Confidence interval			95% Confidence interval		
Pair 1	Weight	Lower	Upper	Loading	Lower	Upper	*p* a
**Age at scan**	**0.90**	**0.84**	**1.02**	**0.98**	**0.96**	**0.98**	**<0.001**
**CPAP (days)**	**−0.10**	**−0.16**	**−0.05**	**0.34**	**0.28**	**0.43**	**0.045**
Gestational age at birth	−0.34	−0.41	−0.31	−0.32	−0.41	−0.27	0.060
Mechanical ventilation (days)	−0.08	−0.14	−0.01	0.29	0.24	0.38	0.101
Birth weight	0.17	0.11	0.22	−0.24	−0.33	−0.19	0.16
Surfactant administration	−0.05	−0.10	−0.01	0.23	0.19	0.30	0.17
**Pair 2**							
**Birth weight**	**0.98**	**0.87**	**1.02**	**0.93**	**0.90**	**0.95**	**<0.001**
**Gestational age at birth**	**−0.01**	**−0.12**	**0.04**	**0.78**	**0.72**	**0.82**	**<0.001**
**CPAP (days)**	**−0.35**	**−0.41**	**−0.28**	**−0.67**	**−0.72**	**−0.61**	**<0.001**
**Parenteral nutrition (days)**	**−0.12**	**−0.18**	**−0.05**	**−0.55**	**−0.63**	**−0.49**	**0.010**
**Surfactant administration**	**0.02**	**−0.03**	**0.08**	**−0.51**	**−0.56**	**−0.43**	**0.002**
**Mechanical ventilation (days)**	**−0.09**	**−0.15**	**−0.03**	**−0.49**	**−0.56**	**−0.42**	**0.01**
**Pair 3**							
**Male sex**	**0.74**	**0.63**	**0.79**	**0.76**	**0.71**	**0.81**	**<0.001**
**IUGR**	**0.20**	**0.12**	**0.24**	**0.42**	**0.32**	**0.47**	**0.011**
No maternal breast milk	−0.08	−0.13	−0.03	−0.23	−0.29	−0.17	0.180
Emergency Caesarean	0.22	0.15	0.25	0.21	0.13	0.26	0.227
Elective Caesarean	0.17	0.10	0.21	0.19	0.11	0.25	0.253
Maternal breast milk and formula	0.09	0.02	0.13	0.17	0.10	0.23	0.304
**Pair 4**							
**IUGR**	**0.06**	**0.01**	**0.16**	**0.50**	**0.42**	**0.56**	**0.002**
**Pregnancy‐associated hypertension**	**0.08**	**0.05**	**0.17**	**0.47**	**0.43**	**0.54**	**0.004**
**Gestational age at birth**	**0.81**	**0.77**	**0.93**	**0.43**	**0.35**	**0.54**	**0.010**
**Male sex**	**−0.19**	**−0.31**	**−0.16**	**−0.38**	**−0.47**	**−0.31**	**0.025**
Elective Caesarean	0.05	0.01	0.11	0.32	0.27	0.39	0.058
CPAP (days)	−0.20	−0.29	−0.17	−0.32	−0.42	−0.24	0.062
**Pair 5**							
**Mechanical ventilation (days)**	**0.79**	**0.68**	**0.87**	**0.64**	**0.56**	**0.70**	**0.005**
**Parenteral nutrition (days)**	**0.11**	**0.01**	**0.18**	**0.43**	**0.34**	**0.51**	**0.029**
**Surgery for NEC**	**0.06**	**−0.04**	**0.16**	**0.42**	**0.33**	**0.50**	**0.043**
Emergency Caesarean	−0.14	−0.19	−0.08	−0.31	−0.37	−0.25	0.066
Male sex	0.29	0.20	0.33	0.28	0.19	0.35	0.103
Nonsingleton birth	−0.33	−0.37	−0.25	−0.27	−0.33	−0.19	0.118

a 10,000 permutations, significant loadings highlighted in bold.

CPAP = continuous positive airway pressure; IUGR = intrauterine growth restriction; NEC = necrotizing enterocolitis.

Below, we describe the relationship between the multimodal imaging markers and their associated clinical variables. To examine the anatomical features of each marker in detail, readers are encouraged to explore the online multimodal interactive maps using Neurovault.

### Age and Prematurity

Figure 2A illustrates the relationship between imaging markers of brain development and age. Age at scan is strongly correlated (*r* = 0.94) with the imaging variate, which is expressed as linked increases in gray matter volume and white matter FA, decreases in gray matter diffusivity, and a pattern of T2 signal intensity change including decreased white matter and subcortical gray matter signal and increased signal intensity in the medial temporal lobe. Clinical loadings in Table 3 demonstrate that age at scan captures the majority of variance in the first clinical variate (canonical loading = 0.98; 95% CI [0.96, 0.98]); however, this pattern of normal growth was slightly reduced by the need for more intensive care (longer ventilation, canonical weight = –0.08 [–0.14, –0.01]) after delivery.

Figure 2B shows the relationship between the second imaging variate and associated clinical factors, with the most highly explanatory variate, birth weight, shown separately. The clinical factors are shown in Table 3 and reflect a pattern of *increasing* maturity at birth: increasing birth weight and gestational age (canonical loadings = 0.93 [0.90, 0.95], 0.78 [0.72, 0.82], respectively) with concomitant negative associations to factors reflecting an increased need for intensive care. These factors were associated with a set of imaging markers comprising increased frontotemporal volume, decreased T2 signal in the basal ganglia, and a smaller interhemispheric fissure (represented by decreased T2 signal in regions occupied by cerebrospinal fluid [CSF]), alongside increased anisotropy in central white matter tracts and localized reductions in mean diffusivity in the thalamus.

### Sex

**Figure 4:**
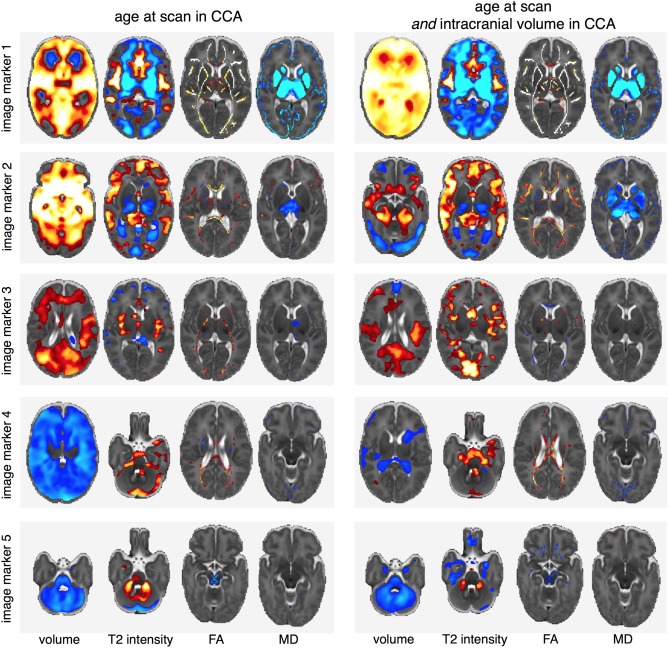
Comparing imaging markers with and without the addition of intracranial volume to the CCA analysis. The addition of ICV to the model results in an additional canonical pair (not shown) detailing the relationship between each modality and total brain volume. As subsequent canonical pairs are defined independent of this relationship, the right column shows each imaging marker corrected for ICV. CCA = canonical correlation analysis; FA = fractional anisotropy; ICV = intracranial volume; MD = mean diffusivity.

Figure 3A illustrates the third canonical pair. Table 3 shows that the strongest contributing clinical factor was sex (loading = 0.76 [0.71, 0.81]), with a small contribution from IUGR (0.42 [0.32, 0.47]). Birth by emergency Caesarean (0.21 [0.13, 0.26]) correlated positively, and formula feeding without maternal breast milk (–0.23 [–0.29, –0.17]) correlated negatively with this factor. This clinical variate was associated with imaging markers comprising increased volume in medial temporal, parietal, and superior frontal regions, the cerebellum and brainstem, alongside increased anisotropy in the internal capsule and localized decreases in gray matter diffusivity. We found that IUGR was marginally more prevalent in males than females (17.7% and 11.7%, respectively; chi‐square = 3.32; *p* = 0.07), though the rates of emergency Caesarean and formula‐only feeding were similar (males, 54.0% and 14.2%; females, 58.7% and 14.8%). When additionally correcting for intracranial volume (ICV; Fig 4), the volumetric differences were reduced and increased T2 signal intensities localized to the insula and primary visual cortex.

### Intrauterine Compromise and Growth Restriction

The fourth canonical pair was positively associated with intrauterine compromise including IUGR (loading = 0.50 [0.42, 0.56]) and maternal hypertension (0.47 [0.43, 0.54]), alongside increasing gestational age (0.43 [0.35, 0.54]; Table 3) and delivery by elective Caesarean section (0.32 [0.27, 0.39]), factors associated with increasing exposure to the intrauterine environment. These factors were associated with a global decrease in brain volume, alongside increased T2 signal intensity suggestive of increased CSF volume apparent in the fourth ventricle and surrounding the brainstem and cerebellum, decreased T2 signal intensity in the lateral ventricles, and localized increases in FA in the corpus callosum (Fig 3B). Independent of the relationship with total brain volume, the lateral ventricles remained significantly smaller (Fig 4), whereas T2 signal intensity remained high in the CSF surrounding the brainstem and FA remained elevated in the corpus callosum.

### Neonatal Sickness

The final significant canonical pair revealed a relationship, independent of preceding pairs, between markers of postnatal sickness (length of mechanical ventilation, length of parenteral nutrition, and surgery for necrotizing enterocolitis) and a striking set of imaging markers including: reduced brain volume, which was most prominent in the cerebellum and brainstem and spared primary cerebral cortex; modest reductions in FA localized to the brain stem and corpus callosum; and increased T2 signal intensity in the cerebellum (Fig 3C). These imaging markers were robust to the inclusion of ICV in the CCA model (Fig 4). Two clinical factors suggested antenatal compromise correlated negatively with this phenotype: birth by emergency Caesarean (loading = –0.31 [–0.37, −0.25]) and a nonsingleton pregnancy (–0.27 [–0.33, −0.19]).

### Neurodevelopmental Outcome

For the 425 infants where neurodevelopment assessments were available, mean (±standard deviation) BSID‐III cognitive, language, and motor scores were: 92.2 ± 13.6, 90.3 ± 17.2, and 94.4 ± 12.8, respectively. Linear associations between imaging variates and outcome scores are shown in Table 4. Cognitive outcome was significantly associated with imaging marker 2. Motor outcome was significantly associated with both imaging markers 2 and 5. Language outcome correlated with image markers 1 and 2, but this did not pass correction for multiple comparisons. Because the imaging and clinical variates correlate strongly within each CCA pair, these reported associations are also present when using the respective clinical variate score instead of the imaging marker.

**Table 4:** Associations Between Multimodal Imaging Markers and Neurodevelopmental Outcome

	Cognitive		Motor		Language	
**Image Marker**	***F*_(1,424)_**	***p***	***F*_(1,424)_**	***p***	***F***_(1,424)_	***p***
1	5.006	0.026	3.265	0.071	4.690	0.031
2	**10.498**	**0.001** a	**18.790**	**<0.001** a	4.927	0.027
3	0.026	0.872	0.268	0.605	2.796	0.952
4	0.502	0.479	0.339	0.561	2.251	0.134
5	2.298	0.130	**7.847**	**0.005** a	2.707	0.101

a Significant at *p* < 0.05 after correcting for multiple comparisons.

### Other Considerations

To investigate whether these relationships were dependent on overall brain volume, we performed CCA with the addition of ICV as a clinical factor. This resulted in an additional canonical pair (canonical correlation, 0.94 [0.90, 0.95]; *p* < 0.001) with a strong contribution of ICV (loading = 0.76 [0.70, 0.78]) to the clinical variate and imaging markers comprising increased anisotropy, decreased diffusivity, and localized (relative) decreases in volume and T2 signal. Figure 4 illustrates the imaging patterns associated with the remaining canonical pairs in comparison to the original markers described above. With inclusion of ICV as a clinical factor, and thus correction of subsequent canonical pairs for associations with age *and* ICV, the imaging patterns associated with pairs 1 and 2 remain apparent across all modalities, with the exception that the volumetric increase associated with increasing birth weight is restricted to the medial temporal lobe in pair 2 when controlling for total brain volume.

CCA is a parametric analysis and so may be sensitive to outliers or non‐normally distributed data. We therefore performed CCA with several alternative strategies accounting for: neonates with MR‐apparent cerebral pathology (eg, intraventricular hemorrhage, cystic lesions) by performing CCA with imaging components clearly relating to pathology removed, or by removing these infants (n = 37) from the analysis entirely; non‐normally distributed clinical data (by applying cubic root, square root, or square transformations as required) before CCA; and, additionally, removed all categorical clinical factors where the number of positive cases <10%. These strategies did not significantly alter the clinical‐image relationships reported above. Additionally, when ignoring lesion cases (n = 37) in the linear regression model, the reported statistical associations with neurodevelopmental outcome remained significant (*p* < 0.05).

## Discussion

We performed a data‐driven, multivariate analysis of a large, prospective, multimodal imaging data set and identified a novel set of alterations in brain structure and microstructure that varied in line with perinatal, clinical, and environmental risk factors. The results showed expected relationships between brain development and age, as well as confirming and extending the range of abnormalities associated with increasing prematurity. Separate from these effects, the analysis revealed unexpected patterns of abnormality associated with sex and antenatal and postnatal adversity, a number of which were associated with neurodevelopmental outcome at 2 years corrected age. We have provided interactive multimodal statistical maps to allow researchers to explore these rich data in detail, and here we offer an initial appreciation of the clinical‐anatomical correlations.

It is well established that brain growth and development during the preterm period is characterized by increases in brain tissue volume and white matter FA, alongside decreasing cortical diffusivity and T2 signal intensity.^7^, ^8^, ^31^, ^32^ We found a single imaging variate that reflected these changes across micro‐ and macrostructural scales. This normal pattern of growth and development was impaired by respiratory illness and nutritional deficits, factors known to affect somatic growth during the neonatal period. Another set of multimodal imaging markers corresponded largely to sex, with male sex associated with volumetric increases in the temporal lobes, medial parietal regions and cerebellum, and increased FA in the internal capsule; and, when corrected for ICV, increased T2 signal intensity primarily in the primary visual cortex. Cortical T2 signal intensity is dependent on numerous developmental processes, including cellular packing and myelination,^33^ and a lower gray matter T2 signal at this stage of development is indicative of more‐advanced maturation.^34^ This indicates a sexual dimorphism in cortical maturation, which may also be related to differences in cortical surface area over the same time period.^9^

Independent of these relationships, we found a pattern of variation reflecting the impact of low birth weight and earlier exposure to the extrauterine environment. In previous studies of similar cohorts, we and others have observed decreased frontotemporal volume, increased thalamic diffusivity, and decreased FA in the central white matter with increasing prematurity.^7^, ^10^, ^12^, ^35^ Here, we show these changes are coupled, independent of age at scan and total intracranial volume, and exacerbated by comorbid markers of neonatal sickness. When additionally controlling for ICV, the effect of birth weight was most apparent in the medial temporal lobe. Reduced hippocampal volume is associated with white matter injury in preterm infants at term‐equivalent age and correlated with later working memory deficits.^36^ This neuroimaging marker is compatible with histological evidence of decreased white matter volume and tissue loss in the cortex, basal ganglia, and hippocampus from primate animal models of preterm birth.^37^ Less premature birth in this study is also associated with a lower T2 signal intensity in the basal ganglia. In a recent survey of the MR correlates of pathology in animal models, a loss or delayed appearance of mature oligodendrocytes throughout the white matter was found to correlate with reduced white matter anisotropy, and increased T2 hyperintensity was correlated to regions of astrocytosis.^38^ In the absence of severe injury, a similar pattern of hyperintensity may occur through the delay of normal myelination, or premyelination, processes.^39^ However, diffuse T2 signal alterations in the white matter, one of the first detected and widely reported abnormalities in the preterm brain,^40^ were not a feature of this imaging phenotype. This is an important consideration for experimental studies focusing on white matter injury in prematurity, given that this endophenotype appears to capture a multifactorial brain injury, with coexistent cellular injury and delayed developmental processes across scales and tissue types that was strongly related to adverse neurocognitive outcomes. This, in turn, suggests that studies focusing solely on white matter development in prematurity may not capture the entire neuropathological picture.

The fourth set of imaging markers comprised a decrease in brain tissue volume linked with increased T2 signal intensities around the cerebellum and brainstem. This was associated with long‐term intrauterine complications, including IUGR and maternal hypertension. IUGR results from placental insufficiency, increases risk for adverse outcome in preterm infants, and, on occasion, may share a common pathogenesis with maternal hypertension.^14^, ^41^ It has previously been difficult to dissociate adverse antenatal and postnatal correlates of abnormal brain development. Here, we demonstrate a separable pattern of development in preterm infants with IUGR marked by global brain growth failure, with additional alterations in the brainstem. Interestingly, this becomes more apparent with increasing gestational age at birth and is also associated with delivery by elective Caesarean, reflecting a clinical decision to deliver a compromised foetus. When correcting for total ICV, volumetric reductions were restricted to the lateral ventricles, but T2 signal intensity around the brainstem remained high. Because of the large contrast between brain tissue and CSF intensities in these regions, small decreases in volume that may not be apparent in the volumetric maps could result in an increase in tissue signal intensity attributed to partial volume effects. However, this finding remains inconclusive given that, although ventriculomegaly has been shown to be less prevalent in small for gestational age (SGA) adolescents compared to those born preterm,^42^ there is only limited evidence of a thinner mesencephalon in SGA preterm infants^43^ and IUGR appears to be related to a relatively larger brainstem and cerebellar volumes in infants born at term.^44^ Increases in callosal FA have been previously reported in growth‐restricted infants at 12 months.^45^

The final canonical pair‐related markers of postnatal sickness, including the need for mechanical ventilation, continuous positive airway pressure (CPAP), and parenteral nutrition with a pattern of brain injury characterized by reduced tissue volume, decreased FA in the brainstem and corpus callosum and increased cerebellar T2 signal intensity. Notably, this pattern was independent of preceding clinical‐image relationships and also total ICV. This multimodal imaging marker was striking in the relative sparing of primary cortex, which may reflect the relative maturity of primary cortex at this age,^8^ and in the changes in diffusivity in the lentiform nuclei, which may reflect altered cell density. Animal models of preterm birth have shown that prolonged respiratory ventilation is associated with adverse cerebral outcomes, including tissue volume loss and white matter injury,^46^ and we have previously demonstrated global volume losses and alterations to white matter microstructure in infants with chronic lung disease.^35^, ^47^ The preterm cerebellum is also specifically vulnerable to perinatal hypoxic‐ischemic events, resulting in high T2 signal and atrophy.^48^

We found that cognitive and motor outcomes were significantly correlated with imaging patterns associated with pairs 2 and 5, which is consistent with robust clinical data showing that prematurity and postnatal sickness are strongly correlated with poor neurodevelopmental outcome.^2^, ^3^ These results provide new insights into the nature of preterm brain injury, and the association of these imaging markers suggests that these abnormalities may be the most important in producing neurodevelopmental impairment.

These associations do not imply causal relations. Each clinical variate is a combination of multiple clinical factors, many of which are correlated, making it difficult to ascribe any effects to a single factor. In this report, we highlight the clinical factors with the largest variate loading as representative of the environmental influence on imaging phenotype. An alternative could be to use methods that enforce sparsity among contributing factors,^49^ although the degree of sparsity required is unknown and both study and context dependent. To derive imaging phenotypes, we used linked ICA to decompose MRI data into a set of linked imaging components. This approach represents a natural framework to capture the wide range of alterations across tissue types previously reported in preterm infants. In linked ICA, there are no assumptions placed on intermodal spatial correspondence^27^; co‐occurring, overlapping spatial patterns across modalities are driven by variance across subjects and can reflect different aspects of a shared physiological etiology. This approach allows the simultaneous estimation of developmental patterns across modalities, a process more amenable to interpretation than combining estimates over multiple, parallel univariate analyses.

A notable omission in the current analysis is the inclusion of genetic data; it is likely that the interaction of environmental and genetic influences will significantly impact brain development following preterm birth. Several advances have been made recently in the joint analysis of imaging and genetic information within joint, or linked, ICA frameworks.^50^ Using similar methods in this cohort could reveal a genetic underpinning to susceptibility for some imaging phenotypes or environmental impacts and represents an important future avenue for investigation. In addition, the accurate ascertainment and diagnosis of infection and sepsis is difficult in preterm infants, and it is possible that this has led to an underestimation of the role of infection in abnormal brain development.

In summary, we combine multimodal neuroimaging with data‐driven multivariate statistical analysis to show how environmental factors give rise to separable developmental trajectories through the cumulative expression of distinct imaging patterns in preterm‐born individuals, and provide a resource for researchers to explore these neuroanatomical features in detail.

## Author Contributions

Study design: A.D.E., N.K., M.A.R., and S.J.C. Acquisition and analysis: G.B., P.A., P.N., S.F., A.C., N.H., S.J.C., N.G.‐C., and J.W. Manuscript and figure preparation: G.B., S.J.C., and A.D.E.

## Potential Conflicts of Interest

Nothing to report.
